# Engagement with consumer smartwatches for tracking symptoms of individuals living with multiple long-term conditions (multimorbidity): A longitudinal observational study

**DOI:** 10.1177/26335565211062791

**Published:** 2021-11-30

**Authors:** Syed Mustafa Ali, David A Selby, Kazi Khalid, Katherine Dempsey, Elaine Mackey, Nicola Small, Sabine N van der Veer, Brian Mcmillan, Peter Bower, Benjamin Brown, John McBeth, William G Dixon

**Affiliations:** 1 Centre for Epidemiology Versus Arthritis, Division of Musculoskeletal and Dermatological Sciences, Manchester Academic Health Science Centre (MAHSC), 5292 University of Manchester, Manchester, UK; 2 Centre for Health Informatics, Division of Informatics, Imaging and Data Sciences, Manchester Academic Health Sciences Centre, 523398 University of Manchester, Manchester, UK; 3 NIHR School for Primary Care Research, Centre for Primary Care and Health Services Research, Division of Population Health, Health Services Research and Primary Care, School of Health Sciences, Faculty of Biology, Medicine and health, Manchester Academic Health Science Centre Manchester, 5292 University of Manchester, Manchester, UK; 4 NIHR Policy Research Unit for Older People and Frailty, Centre for Primary Care and Health Services Research, Division of Population Health, Health Services Research and Primary Care, School of Health Sciences, Faculty of Biology, Medicine and health, Manchester Academic Health Science Centre Manchester, 5292 University of Manchester, Manchester, UK; 5 NIHR Manchester Biomedical Research Centre, 5293 Manchester NHS Foundation Trust, Manchester, UK; 6 7047 Salford Royal NHS Foundation Trust, Salford, UK

**Keywords:** Multiple long-term conditions (multimorbidity), smartwatch, patient-generated health data, user engagement

## Abstract

**Introduction:**

People living with multiple long-term conditions (multimorbidity) (MLTC-M) experience an accumulating combination of different symptoms. It has been suggested that these symptoms can be tracked longitudinally using consumer technology, such as smartphones and wearable devices.

**Aim:**

The aim of this study was to investigate longitudinal user engagement with a smartwatch application, collecting survey questions and active tasks over 90 days, in people living with MLTC-M.

**Methods:**

*‘Watch Your Steps*’ was a prospective observational study, administering multiple questions and active tasks over 90 days. Adults with more than one clinician-diagnosed long-term conditions were loaned Fossil® Sport smartwatches, pre-loaded with the study app. Around 20 questions were prompted per day.

Daily completion rates were calculated to describe engagement patterns over time, and to explore how these varied by patient characteristics and question type.

**Results:**

Fifty three people with MLTC-M took part in the study. Around half were male ( = 26; 49%) and the majority had a white ethnic background (*n* = 45; 85%). About a third of participants engaged with the smartwatch app nearly every day. The overall completion rate of symptom questions was 45% inter-quartile range (IQR 23–67%) across all study participants. Older patients and those with greater MLTC-M were more engaged, although engagement was not significantly different between genders.

**Conclusion:**

It was feasible for people living with MLTC-M to report multiple symptoms per day over 3 months. User engagement appeared as good as other mobile health studies that recruited people with single health conditions, despite the higher daily data entry burden.

## Introduction

Multiple long-term conditions (multimorbidity) (MLTC-M) is defined as having two or more long-term conditions at the same time.^1^ In line with a rising MLTC-M prevalence worldwide,^2^ the proportion of those aged over 65 with MLTC-M in England is projected to increase from 54% in 2015 to 68% in 2035.^3^ One in three emergency hospital admissions have five or more long-term conditions, up from one in ten a decade ago.^4^ Multiple long-term conditions (multimorbidity) reduces quality of life and increases the likelihood of hospital admission, re-admissions and increases overall healthcare costs.^5,6^

People living with MLTC-M have to deal with an accumulating combination of different symptoms – the severity of which varies through time – plus the potential harms of multiple treatments.^7^ Managing one health condition can exacerbate another, and the dynamic nature of symptoms makes it difficult to choose an optimal treatment.^8^ Much research to date is cross-sectional rather than longitudinal, making it impossible to study temporal changes.^1^ Where longitudinal studies do exist, they often measure disease change at widely spaced intervals.^9–11^

Consumer technology, such as smartphones and wearable devices, has been identified as a potential way to track and monitor longitudinal symptoms of people living with MLTC-M.^1^ ‘Bringing together long-term remote monitoring, digital epidemiology and continuous disease monitoring’, and ‘developing measures to collect, link, store and share appropriate data and outcomes for MLTC-M, particularly focusing on longitudinal aspects including continuous disease monitoring’ are included as aims that will drive advances in our understanding of MLTC-M, as stated in the UK’s cross-funder multimorbidity research framework.^12^

Many research studies have successfully used smartphones to track symptoms longitudinally for specific health conditions, such as chronic pain,^13^ rheumatoid arthritis,^14^ heart failure ^15^ and COVID-19.^16^ Smartwatches provide a similarly exciting opportunity for health research, as they combine the ability to self-report symptoms on a wrist-worn touchscreen with passive collection of sensor data, including heart rate and movement.^17,18^ Remote sensor and monitoring technologies, including smartwatches, can record variations in individual’s condition over time, and healthcare professionals can use this information for risk assessment and informed clinical decisions.^19^

An important challenge for MLTC-M using any consumer device, however, is designing a flexible data collection system that can be tailored to measuring symptoms relevant to the specific combination of conditions an individual participant may have. Asking irrelevant questions is likely to increase attrition and drop-out.^20^ There are no published studies where people with multiple long-term conditions used consumer smartwatches to collect multiple symptoms over time. It is thus important to understand whether people living with MLTC-M would be willing and able to track symptoms through time, given their greater burden of disease and treatment (Mair, 2014), and possible lower digital literacy among some older patients (Oh *et al.*, 2021).

Therefore, the aim of this study was to investigate longitudinal user engagement with a smartwatch application, collecting daily survey questions and active tasks over 90 days, in people living with MLTC-M. Specifically, the objectives of the study were to:

1. Describe engagement patterns over time by assessing (a) the completeness of scheduled questions and active tasks and (b) frequency and patterns of unscheduled survey and active task responses;
2. stratify engagement patterns by age, gender, number of disease areas and question type (generic or organ-specific), and by time of day and week;
3. survey participants' views of the acceptability and usability of the smartwatch for data collection.

## Methods

‘*Watch Your Steps*’ was a prospective observational smartwatch study, conducted by the University of Manchester in partnership with Google Fit Research. The study asked people living with MLTC-M to complete multiple daily and weekly questions and active tasks over 90 days. In this section, we first describe the co-design workshop then the data collection system, participant recruitment and data collection procedures before describing our analysis methods to answer each of the three study objectives.

### App co-design workshop with patients, clinicians and researchers

The research team from the University of Manchester specified an initial data collection structure that would include a series of core data items for all participants, complemented with a series of more specific symptoms that would vary according to the disease areas of participants’ different long-term conditions. At a local venue in Greater Manchester, a two-hour multi-stakeholder workshop was attended by people living with MLTC-M (*n* = 6), clinicians (*n* = 6) and researchers (*n* = 6). People living with MLTC-M were recruited from the local patient and public involvement and engagement group. Clinicians and researchers were representatives of the local clinical and research teams from where participants of the *Watch Your Steps* study were recruited. The workshop established a consensus on what generic and organ-specific symptom questions to collect and how often, while balancing the relevance of symptom questions against participants’ burden of symptom reporting. Table S1 (in supplementary material) displays the final list of data items and when and how often they were prompted.

### App development and testing

The Google Fit Research team developed the *Watch Your Steps* study app. Each study participant was prompted (by a notification with an audible vibration) at the specified day/time to complete one of three smartwatch tasks: (i) core symptom questions (for all participants), (ii) organ-specific questions (based on participants’ disease areas) and (iii) active tasks, including a sit-to-stand test, walk test and tap test (see Table S2 of supplementary materials). Questions remained active on the watch face for three hours for daily questions and for 24 hours for weekly questions. For example, a participant received a prompt on their smartwatch to answer the pain level question at 18:00, which then remained active until 21:00. In addition to when prompted, participants could answer any question at any time through the app menu, including those not required for their baseline disease areas.

**Figure 1.**
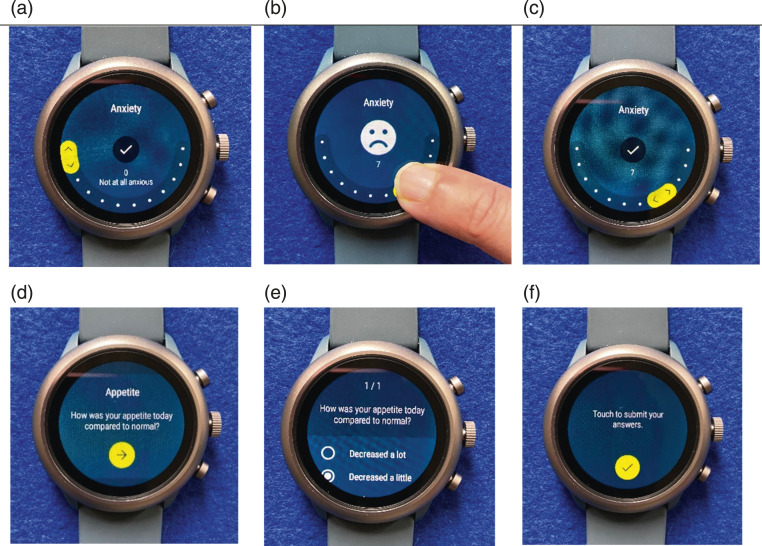
Images of the smartwatch face showing different input methods and their steps. (a) Radial interface for anxiety (a symptom question with a numerical rating scale response). (b) Moving selector on the radial interface showing a dynamic emoticon. (c) Submitting response by tapping the tick mark. (d) Wording of the appetite question (a symptom with a categorical response). (e) Selection of a categorical response option. (f) Submitting response by tapping the tick mark.

Fossil Sport smartwatches were pre-loaded with the study app (Figure 1) and loaned to participants for the duration of the study. Each watch had a unique code assigned to the study participant. Participants were also provided with a mobile broadband router (or Mi-Fi device). They were advised to dock their watch each night for charging, at which time the encrypted study data would also be transmitted securely. This information along with guidance on data collection via watch face and contact details for troubleshooting support were included in the user guide (annexure S1: supplementary materials).

Responses to questions were collected either as a numerical value 0–10 by moving a selector around a radial interface (Figure 1(a)–(c)), or as categorical responses (Figure 1(d)–(e)). Questions with a numeric rating scale included word anchors at 0 and 10 (e.g. no pain and worst possible pain; supplementary Table S1) and a dynamic emoticon that varied to illustrate ‘good’ vs ‘bad’ responses (Figure 1(b)). The smartwatch also collected continuous passive data on physical activity and heart rate from its gyroscope, accelerometer and photoplethysmography (detects volumetric changes in blood) sensors.

The smartwatch did not support any other application in addition to the *Watch Your Steps* data collection app, except displaying the date and time. During the study period, participants did not receive any feedback or summary of their data via the watch face. However, we shared personalised graphical summaries of the data upon study completion.

### Participant eligibility and recruitment

#### Eligibility criteria

Adults (aged 18 and above) with more than one clinician-diagnosed long-term condition were eligible to take part in the study if they were willing to wear a smartwatch for 90 days and were able to understand written instructions in English. We excluded bedbound patients and those who lacked capacity to provide informed consent.

#### Recruitment

Participants were recruited from rheumatology, dermatology, elderly care, respiratory and renal medicine outpatient departments at a local teaching hospital; one community general practice surgery in Greater Manchester; and two local patient and public involvement and engagement groups.

Potential participants either self-referred in response to study flyers or posters displayed in waiting areas or sent via the patient and public involvement and engagement groups, or were encouraged by clinicians to contact the research team. Interested participants were emailed the participant information sheet and the University of Manchester privacy notice (http://documents.manchester.ac.uk/display.aspx?DocID=37095). A researcher was also available in the waiting area on selected days to facilitate recruitment.

Potential participants were screened by telephone for eligibility by a researcher using a screening proforma. Eligible participants were invited to an on-boarding event, where they were asked to sign the consent form, instructed how to use their smartwatch and provided with a copy of the app user guide. Recruitment and on-boarding were done remotely via telephone or Zoom following the onset of the COVID-19 pandemic.

### Data collection

Data collection was started in December 2019 and continued during the COVID-19 pandemic and completed in September 2020. As part of their on-boarding, participants completed a web-based baseline questionnaire to record their demographics and their existing use of health apps (annexure S2: supplementary materials). Participants also completed an introductory questionnaire on the study smartwatch about disease areas and employment status to guide subsequent questions (see Table S1: supplementary materials).

Participants were prompted to complete daily and weekly smartwatch surveys and active tasks as described above. Upon completion of the 90 days’ study period or withdrawal from the study (if they had an experience of more than one day of data collection), study participants were invited to complete a web-based end-of-study questionnaire to assess acceptability and usability of the smartwatch data collection (annexure S3: supplementary materials).

### Data analysis

**Figure 2.**
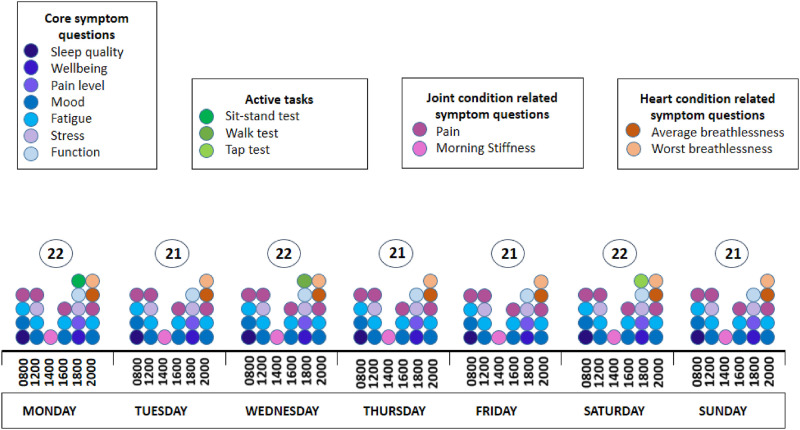
Daily burden of data collection of a hypothetical participant. For this participant, the number of daily scheduled questions is constant at 21 per day with three additional active tests through the week, that is, sit-to-stand test on Monday, walk test on Wednesday and tap test on Saturday, totalling 150 scheduled tasks per week.

Responses were classified as either scheduled or unscheduled. Completion of scheduled responses occurred when they were provided for ‘the question at the right time’, that is, generic or organ-specific questions or tasks were answered within the allocated time window. For a given interval, a participant’s *completion rate* was the number of responses received to unique scheduled tasks, divided by the total number of tasks scheduled during that period. So, for example, consider a hypothetical participant who reported having a joint condition and a heart condition. On a given Monday during the study period, they would be asked to answer 14 ‘generic’ questions, one active test (sit-to-stand), plus seven additional organ-specific questions (including stiffness, pain and breathlessness), amounting to 22 scheduled tasks (figure 2). The completion rate for that day is the proportion of those 22 tasks (21 questions and 1 active test) for which at least one response was received within the respective time windows.

Users could also provide responses that were either to the ‘wrong questions’ (not relevant to their baseline health conditions) or at the ‘wrong time’ (outside the scheduled time window) or both, by accessing these tasks via the in-app menu. The daily completion rate does not capture such unscheduled or additional responses. The distribution of the frequency and timing of additional tasks for each participant were summarised via density plots and dot plots.

### Objectives 1 and 2: Comparing engagement patterns over time, overall and compared between groups

Engagement patterns over time are represented graphically via longitudinal line graphs of daily completion rate, aligned on the first day of each participant’s involvement, to provide an idea of attrition or dropout throughout the 90 days. The trajectory of these lines – their peaks and troughs – offer an indication of how many scheduled responses the participants provided on each day of the study, while allowing for different participants answering different questions according to their respective health conditions. The distribution of overall completion rates is summarised via dot plots.

Grouping these visualisations by participants’ sex, age group, number of disease areas and employment status, we can see visually if there appear to be systematic differences in how different groups engaged with the study.

### Objective 3: Acceptability and usability of the app

The responses collected through web-based end-of-study questionnaire were analysed descriptively and presented as frequencies and percentages.

### Ethical approval

The study received a favourable NHS REC opinion and HRA approval (19/WM/0307).

## Results

### General characteristics of the study participants

Out of 62 people screened for eligibility, 85% (*n* = 53) consented to take part in the study. Around half were male (*n* = 26; 49%) and the majority had a white ethnic background (*n* = 45; 85%) (Table 1). Just over half were aged between 50 and 69 (*n* = 28; 52%); most were employed (*n* = 31; 58%); and the majority reported three or fewer disease areas (*n* = 46; 87%). ‘Bone, joint and muscle’ was the most common disease area (68%) followed by ‘skin’ (45%) and ‘heart and lung’ (40%). Eight people formally withdrew from the study before 90 days due to health problems, perceived side-effects of smartwatch use (e.g. rash) or other reasons.

**Table 1.** Characteristics of the study participants.

Characteristics	Categories	Number (percentage)
Gender	Male	26 (49)
	Female	26 (49)
	Prefer not to say	1 (2)
Age	18–29	9 (17)
	30–39	3 (6)
	40–49	9 (17)
	50–59	14 (26)
	60–69	14 (26)
	70–79	4 (8)
Ethnicity	White	45 (85)
	Mixed or non-white	8 (15)
Employed	Yes	31 (58)
	No	22 (42)
Number of disease areas^a^	1	10 (19)
	2	22 (42)
	3	14 (26)
	4	5 (9)
	5+	2 (4)
Pre-specified list of disease areas	Bone, joint and muscle (e.g. arthritis, neck/back pain, chronic pain)	36 (68)
	Skin (e.g. psoriasis, eczema)	24 (45)
	Heart and lung (e.g. angina, heart failure, COPD/asthma)	21 (40)
	Stomach and bowel (e.g. persistent nausea and vomiting, inflammatory bowel disease)	18 (34)
	Kidney (e.g. chronic kidney disease)	8 (15)
	Endocrine (e.g. diabetes, thyroid disorders)	18 (34)
	Mental health (e.g. anxiety, depression, schizophrenia)	20 (38)
	Neurological (e.g. epilepsy, MS, Parkinson)	8 (15)
Do you own any activity monitoring devices?	Yes	22 (41)
	No	31 (59)
Do you use any smartphone health/well-being apps?	Yes	25 (47)
	No	28 (53)
How frequently do you use any smartphone, smartwatch health/well-being apps?	Always	10 (19)
	Often	9 (17)
	Sometimes	9 (17)
	Never	25 (47)

^a^All participants were confirmed as having two or more LTCs during eligibility screening. The number of pre-specified disease areas refers only to the specific, named organ systems listed here.

### User engagement patterns

#### Completion of scheduled questions

**Figure 3.**
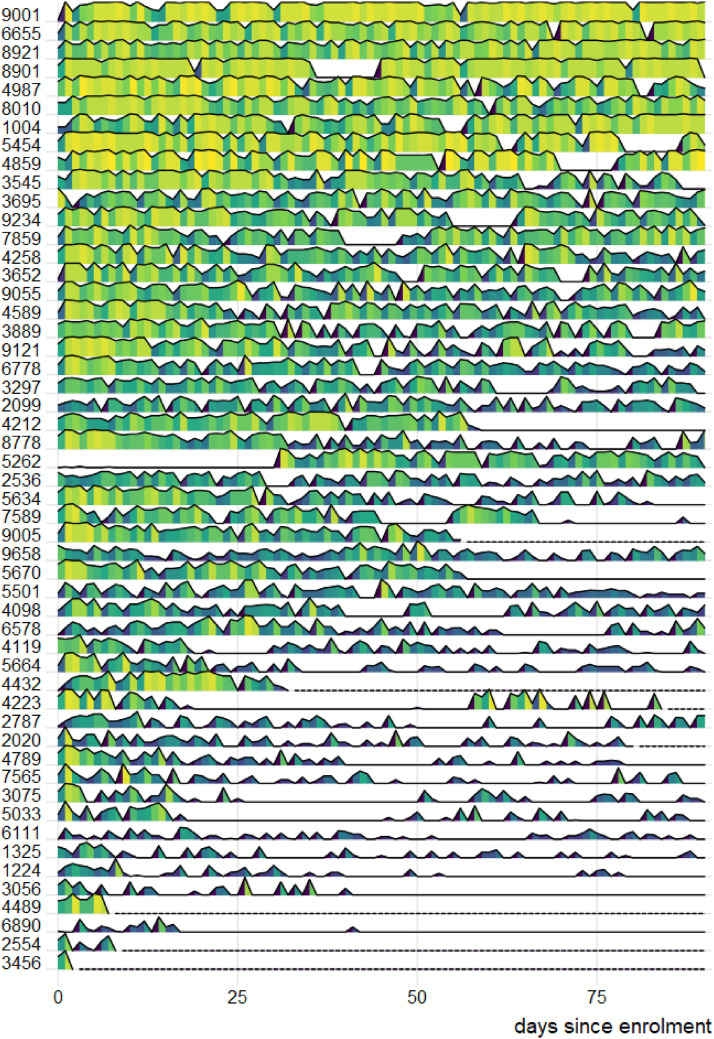
Proportions of scheduled daily questions answered by each participant on each day of the three-month study period. Daily completion rate is encoded by the height and colour: tall, bright yellow segments represent days with nearly 100% of scheduled questions answered by a participant that day. Low and dark blue areas represent low completion, with a flat line indicating zero scheduled responses on a given day. Eight users formally dropped out of the study at which point their line showed as a dotted line. The y-axis labels are four-digit participant identifiers.

Figure 3 shows the daily completion rate over time for all participants. About a third of participants engaged with the app nearly every day of the study period, completing at least one survey response in a day. Over three quarters of the participants (*n* = 41; 77%) stayed in the study by providing data throughout the study period, albeit sometimes at low rates and sporadic intervals. The overall completion rate of symptom questions was 45% (interquartile range (IQR) 23–67%) (see figure 6).

**Figure 4.**
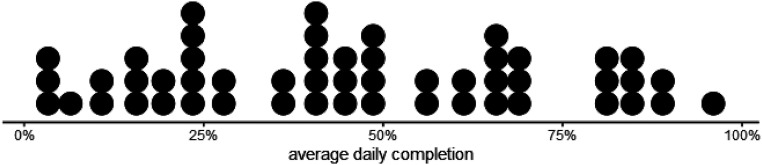
Distribution of average daily completion rate of scheduled questions among the participants over the whole 90-day study period. Each dot represents one participant.

Figure 4 shows the marginal distributions: average engagement per participant over the whole study period. A lower average engagement could be attributed to consistently fewer responses provided each day, or periods of high engagement punctuated by gaps with zero responses.

#### Completion of scheduled active tasks

Participants had 13 unique opportunities to complete each active task – once for each week of the study. None of the participants completed all assigned active tasks; the tap test was completed more often (median 5 out of 13 completed; IQR 2–8) than the sit-to-stand (median 4; IQR 2–7) or walk test (median 3, IQR 2–5).

#### Engagement with unscheduled tasks

**Figure 5.**
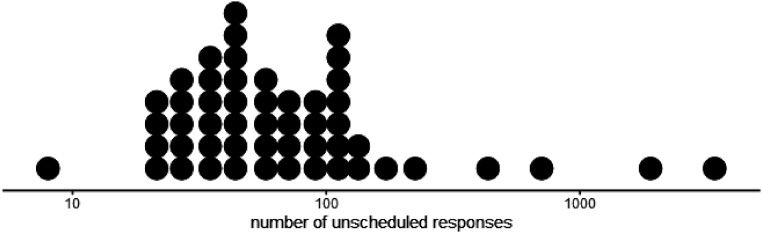
Distribution of the number of unscheduled responses received from each participant over the whole 90-day study period. Each dot represents one participant. Presented on a logarithmic scale.

With no upper bound for the possible number of unscheduled responses a user might provide, there was a greater variation among participants. Figure 5 shows the frequency distribution of unscheduled responses. All participants (except one who did not contribute any data) provided at least one unscheduled response, with two participants providing more than a thousand unscheduled responses in the 90-day period.

‘Additional or unscheduled active tasks’ occurred when participants completed tasks outside the specified 24-hour window. These unscheduled responses were distributed uniformly through the week: there was no particular day that participants appeared to prefer over the ones scheduled.

### Stratified user engagement patterns

Engagement patterns were similar when grouped according to baseline characteristics of the participants (Figure S1: supplementary material). It appeared that many of the most consistently highly engaged participants were over 60 years old, whereas under-30s tended to be either less engaged or less consistently engaged (Figure S1a: supplementary material). People with more than three disease areas were among the most engaged (Figure S1d: supplementary material). Engagement was not different between genders (Figure S2: supplementary materials). There was no obvious difference in engagement pattern between those who use smartphone health/well-being apps (*n* = 25; median 46%; IQR 23–64%) and those who do not use any smartphone-based health/well-being apps (*n* = 28; median 43%; IQR 23–67%). Ridge plots of engagement, stratified by prior use of health/ well-being apps, are included in the online supplementary materials.

**Figure 6.**
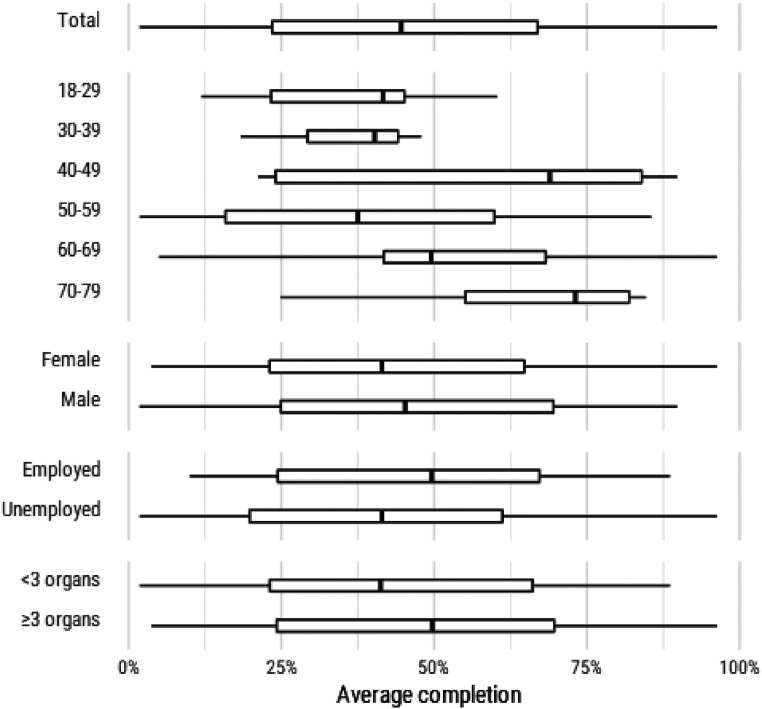
Average proportions of scheduled survey questions completed on time over the study period.

Medians and IQRs of completion rates of scheduled survey questions are provided in figure 6.

Across different disease areas, we found that people with kidney conditions had the highest completion rate (81%; IQR 57–84%) and people with a mental health condition had the lowest completion rate (33%; IQR 17–61%) (see table 2). Ridge plots of engagement, stratified by disease areas, are included in the online supplementary materials.

**Table 2.** Average completion of scheduled survey questions on time per disease area.

Disease area	Total participants (*n*)^a^	Median (IQR) (%)
Bone, joint and muscle	35	45 (24–66)
Skin	22	48 (40–69)
Heart and lung	22	53 (28–79)
Stomach and bowel	20	47 (26–67)
Kidney	7	81 (57–84)
Mental health	20	33 (17–61)

^a^Results represent participants who reported the presence of that disease area, irrespective of other conditions. As this is a study of MLTC-M, participants could be included in more than one group.

**Figure 7.**
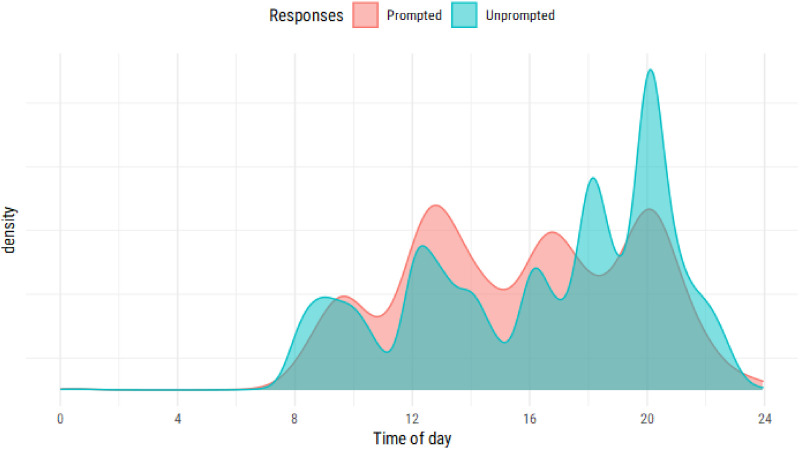
Marginal distribution of responses by time of day. Responses are divided into ‘prompted’ (relevant to a participant’s baseline conditions) and ‘unprompted’ (additional questions the participants answered through the app). Untimely responses are included.

While responses to scheduled questions and active tasks were distributed throughout the day, unscheduled responses were reported more commonly in the evening (Figure 7).

There was no clear difference in the number of unscheduled or additional tasks completed by male versus female participants, nor among those with different numbers of disease areas. But unemployed and older participants were more likely to provide a large number of unscheduled responses compared to employed or younger people, respectively (Figure S3: supplementary materials).

### Usability and acceptability of smartwatch data collection

**Figure 8.**
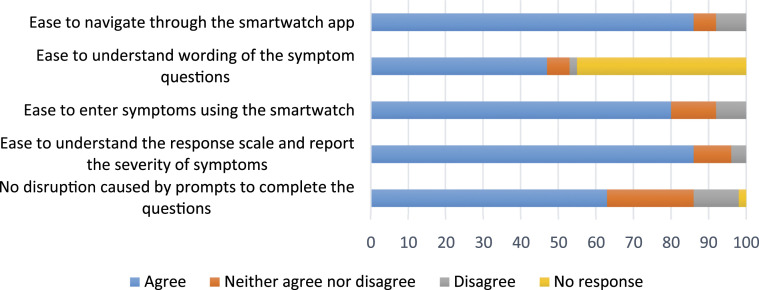
Key usability aspects of the smartwatch study app. Frequency, timing and type of symptom questions.

Out of 53 invited study participants, 49 self-completed the end-of-study questionnaire. Figure 8 shows that the majority of those (strongly) agreed that it was easy to navigate the smartwatch study app (*n* = 42; 86%), while slightly more females (92%) agreed to this than males (80%). They found it easy to understand the response scale (*n* = 42; 86%) and to enter symptoms using the smartwatch (*n* = 39; 80%). In terms of ease of understanding and entering answer to the response scale, there was no significant difference between groups across genders and age groups.

Most participants (*n* = 31; 63%) did not consider data collection tasks disruptive, while slightly more participants with greater disease burden (≥3 disease areas) (5 out of 21; 24%) found it disruptive than participants with lesser disease burden (3 out of 30; 10%). However, almost all (*n* = 48; 98%) stating that the smartwatch did not stop them from going about their normal daily activities (figure 8).

Most study participants (63%; *n* = 31) thought the total number of questions per day was about right. More than half of the study participants found data collection at five time points per day ‘about right’ (*n* = 29; 59%) while around a third (*n* = 15, 31%) felt that it was ‘a bit too high’.

Among those who responded to the questions related to the timings of symptom questions, more people found 12 noon questions convenient to answer (62%), followed by 08:00 (61%), 16:00 (56%), 20:00 (53%) and 18:00 (40%) hours. Moreover, nearly half of the study participants (*n* = 22; 45%) said that they would like to report their symptoms after 20:00 hours.

**Figure 9.**
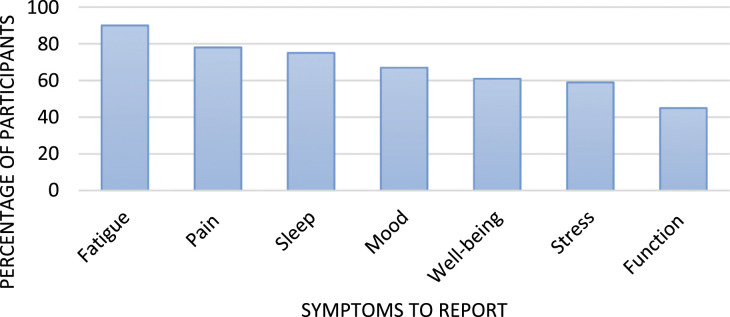
Symptoms participants considered useful to track.

Figure 9 shows that, of the seven core symptoms, fatigue was perceived by most people (*n* = 44; 90%) as a useful symptom to track followed by pain (*n* = 38; 78%) and sleep (*n* = 37; 75%).

## Discussion

### Key findings

We have demonstrated that using a smartwatch for health data collection is feasible and acceptable for individuals living with MLTC-M over a 90-day period. Across all participants, we found a median completion rate of scheduled tasks of 45% (IQR 23–67%), with the highest rate among 70–79 year-olds (73% completion (IQR 55–82%)). Patterns of engagement were heterogeneous; older participants and participants with more than three disease areas were among the most engaged. Despite the high burden of diseases and symptoms and number of questions asked per day, individuals with MLTC-M mostly engaged with the smartwatch data collection system throughout the 90-day period. Whether this level of engagement is acceptable for future research will, of course, depend on what research question is being addressed. Overall, we were pleasantly surprised that the engagement was so high given we were ambitious in asking for over 20 responses per day.

Participants found the app easy to navigate, interact with and understand. The majority felt that the frequency (5–6 times per day) and volume of tasks (14–22 tasks per day) was ‘about right’. Participants reported that 20:00 was the most convenient time at which to be prompted. The same was reflected in the completion rate being highest at 20:00, both for scheduled and unscheduled responses. Though 20:00 appeared to be a suitable time for prompts on the smartwatch, future data collection system should consider offering a customisation feature to participants about selecting type and timing of prompts that suit their self-reporting preferences, assuming that aligns with the data requirements for the study (21). Fatigue, pain and sleep disturbance were rated as the most useful symptoms to track by participants, and active tasks were less likely than survey questions to be completed as scheduled.

### Strengths and limitations

Consumer technology has been recognised as an opportunity for MLTC-M health research, yet most digital health studies were conducted in younger or healthier people.^22^ To our knowledge, this was the first study to examine the use of a smartwatch app designed for people with multiple long-term conditions. We designed a data collection system with key stakeholders to find a balance between utility and reporting burden of multiple daily symptoms which might have helped to sustain engagement during the study. Furthermore, we designed the semi-configurable data collection system using a model of ‘core data’ and ‘data by disease area’, which contrasted with studies that typically asked only disease-specific questions matched to clear recruitment criteria for those diseases.^23^

The study also had some limitations. Study participants self-selected to participate and may therefore not be representative of all patients living with MLTC-M. This motivated cohort may have generated more generous engagement patterns compared to an unselected cohort. The age distribution was younger than we might see in unselected MLTC-M populations.^24,25^ Nonetheless, the results indicated that a larger cohort of people might be willing to participate in a smartwatch study.

The study duration was 90 days, and inferences about engagement patterns beyond this time point cannot be made. Engagement with mobile health (mHealth) applications tends to decay with time, and the potential utility of symptom tracking in both clinical and research contexts might require engagement for a longer interval, depending on the study question.^26^

Previous publications have highlighted that incorporating a mechanism for feedback to users about inputted data can help sustain engagement.^27–29^ The presence of real-time feedback to participants would potentially have improved their engagement in this study.^30^ Motivation may also have been greater if there has been a clear scientific question being addressed that was important to the participants.^28^

### Relation to previous work

Assessments of engagement in previous smartwatch studies relate broadly to adherence with either active data collection or passive monitoring for a single long-term condition. Approaches to defining engagement with active data collection are variable, which makes directly comparing reported rates of engagement challenging.^30,31^ Whilst we had a relatively high burden of daily questions and tasks with no feedback on the watch face, we maintained reasonable levels of engagement compared to other studies.^18,30,31^ The patterns of engagement were similar to our previous smartwatch study in patients with knee osteoarthritis, despite the higher number of questions per day in this study and the difference in populations (MLTC-M versus knee osteoarthritis).^18^ Elm et al. studied 51 participants with Parkinson’s Disease, requiring three patient-reported outcome measures to be entered via an app per day.^32^ By three months, they reported around 45% completion, a figure similar to our MLTC-M population who were responding to around 20 questions per day. The group with highest engagement was older than average in their study, similar to our finding. However, in several web- and smartphone-based studies, as reported in a systematic review, engagement was lower in older people.^31^ Midaglia et al, in their study of 75 individuals aged between 20 and 57 years with multiple sclerosis, defined engagement to scheduled active tasks as the proportion of study weeks with at least 3 days of complete tasks. They observed an overall adherence to active tasks of 70% and remained broadly stable over the 24-week study period.^33^

### Implications for future research

In the present study, prompts were personalised at the level of the disease area. Future digital health studies could be strengthened by more flexible and personalised data collection schedules reflecting the specific conditions affecting an individual, the symptoms that they perceive as being a priority and their lifestyles. We should consider however that increased heterogeneity in responses will complicate comparison between participants. Therefore, these two priorities ought to be balanced carefully depending upon the context and scientific question.

For researchers planning future digital health studies, we hope that our findings will give an indication of the expected levels and patterns of engagement in a cohort of people living with MLTC-M. Our findings will also guide future optimal scheduling of data collection. Greater engagement could be achieved by adding incentives such as feedback of tracked symptoms. In the current study, the sole purpose of data collection was to support research. In the future, integrating patient-reported symptoms (+/- sensor data) from smartwatches into electronic health records could also support clinical care: visual summaries of longitudinal tracked symptoms can provide a clearer picture of disease for better shared decision making.^14,34^

## Conclusion

This study demonstrates that it is feasible for people living with MLTC-M to report multiple symptoms per day over several months. It suggests that participant engagement can be as good as in other mobile health studies that recruited people living with a single health conditions, and which had a lower data entry burden per day. In the future, integrating patient-reported symptoms (+/- sensor data) from smartwatches into electronic health records could also support clinical care by providing visual summaries of longitudinal tracked symptoms for better shared decision making. The study provides evidence that digital epidemiology using personal devices might indeed deliver against its promise^12^ for MLTC-M research.

## Supplemental Material

sj-pdf-1-cob-10.1177_26335565211062791 – Supplemental Material for Engagement with consumer smartwatches for tracking symptoms of individuals living with multiple long-term conditions (multimorbidity): A longitudinal observational study Click here for additional data file. Supplemental Material, sj-pdf-1-cob-10.1177_26335565211062791 for Engagement with consumer smartwatches for tracking symptoms of individuals living with multiple long-term conditions (multimorbidity): A longitudinal observational study by Syed Mustafa Ali, David A Selby, Kazi Khalid, Katherine Dempsey, Elaine Mackey, Nicola Small, Sabine N van der Veer, Brian Mcmillan, Peter Bower, Benjamin Brown, John McBeth and William G Dixon in Journal of Comorbidity

sj-pdf-2-cob-10.1177_26335565211062791 – Supplemental Material for Engagement with consumer smartwatches for tracking symptoms of individuals living with multiple long-term conditions (multimorbidity): A longitudinal observational study Click here for additional data file. Supplemental Material, sj-pdf-2-cob-10.1177_26335565211062791 for Engagement with consumer smartwatches for tracking symptoms of individuals living with multiple long-term conditions (multimorbidity): A longitudinal observational study by Syed Mustafa Ali, David A Selby, Kazi Khalid, Katherine Dempsey, Elaine Mackey, Nicola Small, Sabine N van der Veer, Brian Mcmillan, Peter Bower, Benjamin Brown, John McBeth and William G Dixon in Journal of Comorbidity

sj-pdf-3-cob-10.1177_26335565211062791 – Supplemental Material for Engagement with consumer smartwatches for tracking symptoms of individuals living with multiple long-term conditions (multimorbidity): A longitudinal observational study Click here for additional data file. Supplemental Material, sj-pdf-3-cob-10.1177_26335565211062791 for Engagement with consumer smartwatches for tracking symptoms of individuals living with multiple long-term conditions (multimorbidity): A longitudinal observational study by Syed Mustafa Ali, David A Selby, Kazi Khalid, Katherine Dempsey, Elaine Mackey, Nicola Small, Sabine N van der Veer, Brian Mcmillan, Peter Bower, Benjamin Brown, John McBeth and William G Dixon in Journal of Comorbidity
