# Supplementary material for: Engagement with consumer smartwatches for tracking symptoms of individuals living with multiple long-term conditions (multimorbidity): A longitudinal observational study
Source: J Multimorb Comorb. 2021 Nov 30;11:26335565211062791. doi: 10.1177/26335565211062791 (PMC8637784; doi:10.1177/26335565211062791)
Supplement: sj-pdf-1-cob-10.1177_26335565211062791 – Supplemental Material for Engagement with consumer smartwatches for tracking symptoms of individuals living with multiple long-term conditions (multimorbidity): A longitudinal observational study [file sj-pdf-1-cob-10.1177_26335565211062791.pdf]

Watch Your Steps
Study Smartwatch
User Guide

Contents

Quick Start Guide **pg 3**

Main User Guide

1. Setting up charging and connectivity at home **pg 4**
2. Using the smart watch **pg 6**
3. The *Watch Your Steps* app **pg 8**
4. Completing tasks **pg 10**
5. Understanding surveys and active tasks **pg 12**
6. Frequently asked questions (FAQs) **pg 15**

Contact details **pg 17**

Appendix – app menu of daily and weekly surveys **pg 18**

Quick Start Guide

Our *Watch Your Steps* app aims to track your symptoms multiple times per day. The watch will prompt you to answer surveys at various times through the day, as well as a series of active tasks each week. You can also enter additional surveys whenever you want.
The watch collects continuous heart rate and activity data, so please wear the watch from getting up to going to bed at night. Remember to charge the watch overnight.

1. Get familiar with the smartwatch

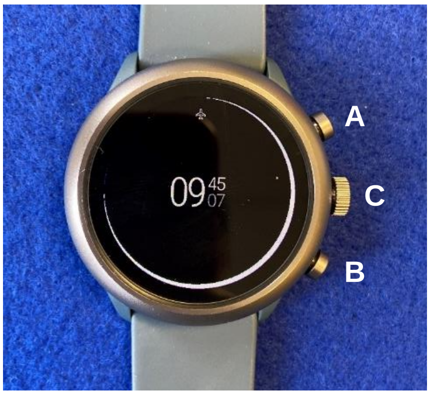

Buttons:
A & B – press button A or B to turn the watch on and open the *Watch Your Steps* app.
C - use this scroll wheel to move the screen up and down. Press button C to go back one step, or keep pressing C to get back to the home screen.

On-screen swiping: Swiping can be used instead of the scroll wheel. Swipe your finger left to right if you want to go back: you can also swipe upwards to see if any tasks are pending.

Sending your study data: Plug the twin USB plug into a wall power socket, then connect the Mi-Fi device to one of the two USB ports. Charge the watch via the other USB port. The watch will automatically connect to the Mi-Fi device during charging and send your study data.

Re-setting: If for any reason the watch stops working, you can try re-setting by pressing and holding down button C for 20 seconds.

2. How to open the *Watch Your Steps* app

When tasks are scheduled to be completed, the watch will buzz and the screen will show you that a task is available to complete. Tasks include 'surveys' and 'active tasks'. You can open the app at any time by pressing button A or B. The home screen will show:
1. The number of outstanding tasks waiting to be completed;
2. The full menu of surveys and active tasks: you can answer any of these whenever you want.

3. How to answer surveys on the smartwatch

There are two ways of answering surveys: a symptom input wheel (0-10), and discrete categories to select from. Having chosen your response, press the **√** button to submit your data.

4. How to do active tasks on the smartwatch

You will be prompted by the watch to do different simple active tasks over the course of a week. These include a **Sit-Stand Test**, **Walk Test** and a **Tap test**. Follow the instructions, and the watch sensors will record your activity. You can decline to do these, or abandon attempts at any time.

Main User Guide

1. Setting up the watch charger and connectivity at home

At the welcome event, you will be given:

1. **A smartwatch** with the *Watch Your Steps* app already installed
2. **A charging cable** for the watch
3. **A MiFi device** with **charging cable** for receiving then transmitting the study data (Figure 1)
4. **A twin USB charger** (Figure 2) to provide power for both the watch and the mifi device.

You should wear the watch throughout the day then charge it overnight. Once the watch is connected to its charger, your data is automatically sent to the MiFi device which then sends it off to the research team. The watch needs to be near the MiFi device for this to work well, so please use the twin USB charger to power both the watch and the MiFi

It is important that you plug both the watch and the MiFi device into the twin USB charger

Setting up the MiFi device:

- Connect the charging lead to the Mi-Fi device
- Plug the other end of the lead into the USB charger ideally positioned near an outside wall – leave this on for the duration of the study
- Once the power is connected correctly the battery level light will go green
- The MiFi device can send the data when the connectivity level is good. The connectivity light is yellow for low and green for good. If the light is red, try moving the device to a different power socket in your home until the light goes green.

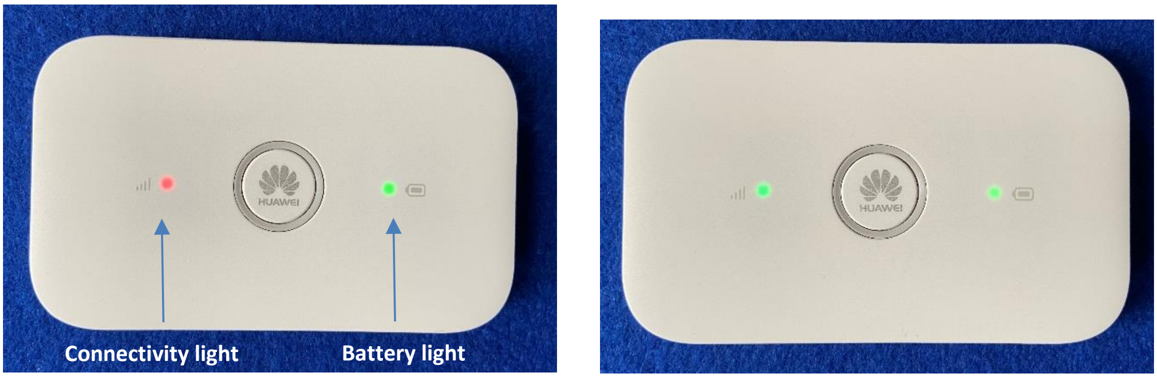

Figure 1. MiFi device. The left image shows poor connectivity (red light) – try moving the device to near an outside wall – the right image shows good connectivity (green light).

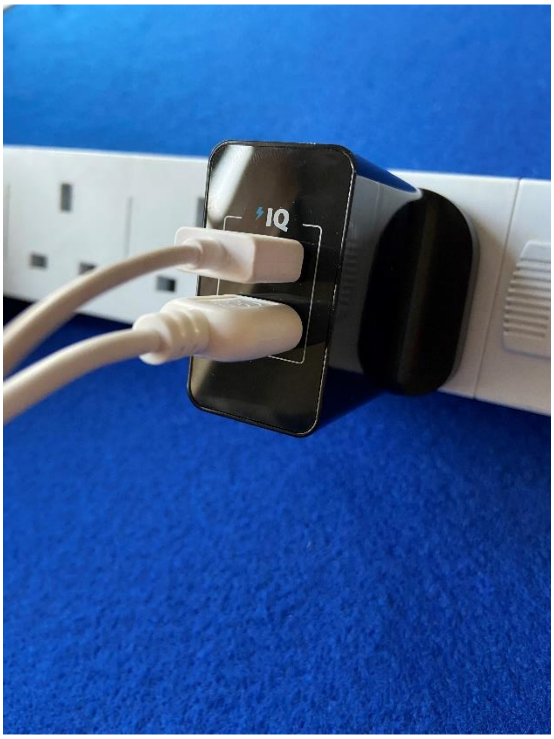

Figure 2. Twin USB charger

To charge the smartwatch:

- Take the smartwatch off your wrist.
- The watch charger has a magnetic end that connects to the metallic bottom of the watch (Figure 3).
- Attach the disc end of your charger cable to the metallic bottom of the watch.
- It connects via the USB cable to the separate twin USB port plug: plug in bottom one.
- A full charge takes between 60-90 minutes – we recommend doing this overnight.
- To avoid damage to your watch only use the magnetic charger provided.
- Depending on use, if charged as directed you should get up to 24+ hours of use.
- The watch will buzz when the battery is low and the battery symbol will show as empty on the watch face.
- If this happens during the day, recharge as soon as you can, then start wearing the watch again until that evening when it should be charged overnight again.
- If the watch doesn't turn on, it may need to be charged

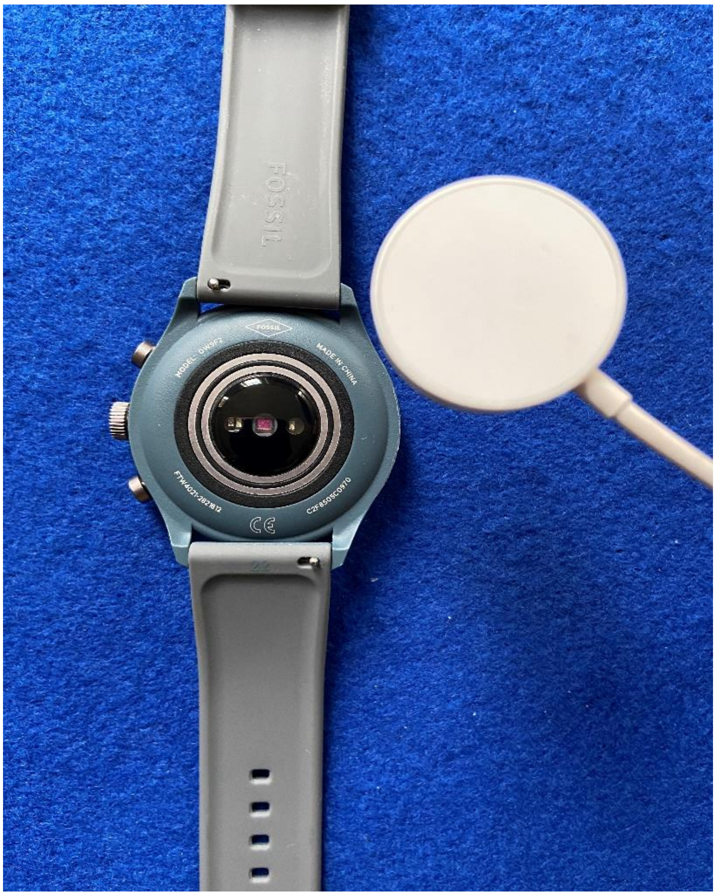

Figure 3. Connecting the smartwatch to its power cable

Top tip: The watch only works when it has battery power left. When you go to bed, always put your smartwatch on charge by plugging it in the second USB twin charger (next to the Mi-Fi). This is also when your data is automatically uploaded to the server via the Mi-Fi device.

2. Using the smart watch

The watch has three buttons A and B and a central button C.

Buttons

A & B – both A & B buttons have the same function. Press button A or B to turn the watch on and open the *Watch Your Steps* app.
C - Press C when on the Rest screen (Figure 4) to see the Home screen. Use this scroll wheel to move the screen up and down. Press button C to go back one step, or keep pressing C to get back to the home screen.

On-screen swiping: Can be used instead of the scroll wheel. Swipe your finger left to right if you want to go back. You can swipe up from the home screen to see if any tasks are pending. You can swipe down from the home screen to put your watch in aeroplane or sleep mode.

Screen views

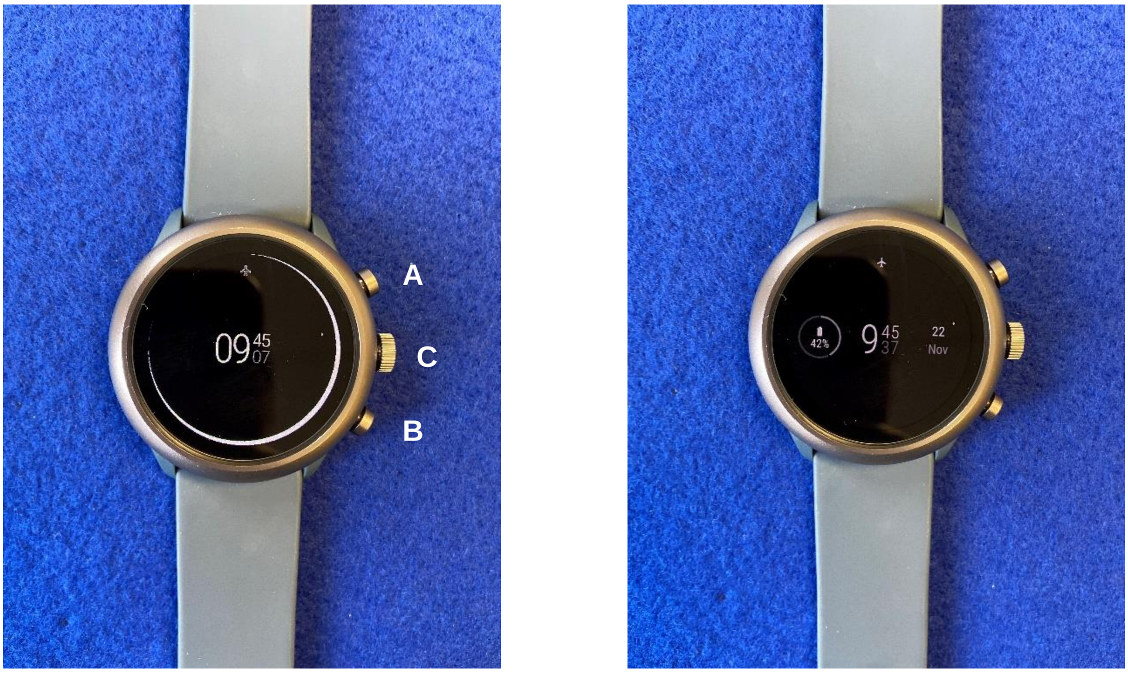

Figure 4. Watch buttons, Rest screen (left) and Home screen (right)

Rest screen (Figure 4, left)

- 24 hour time, minutes and seconds shows when the watch is not in use.
- a symbol at the top of the face shows either an aeroplane during the day, or a WiFi symbol at night when your watch is connected to the Mi-Fi device.
- a small white dot appears at the bottom of the watch face when a task is ready to be completed.

Home screen (Figure 4, right)

Press C to see the Home screen. The home screen shows:

- the 24 hour time, minutes and seconds (centre)
- the date (right hand side)
- the battery life symbol and battery charge percentage (left hand side)

Aeroplane icon and sleep mode

If you swipe down from the home screen, you will see two icons: an aeroplane and watch icon.

- Aeroplane icon: The watch is automatically in aeroplane mode during the day. If you click on this, it will try to pair with the MiFi device.
- Watch icon: Tapping this icon turns the screen off. You can get the screen back on by tapping any button

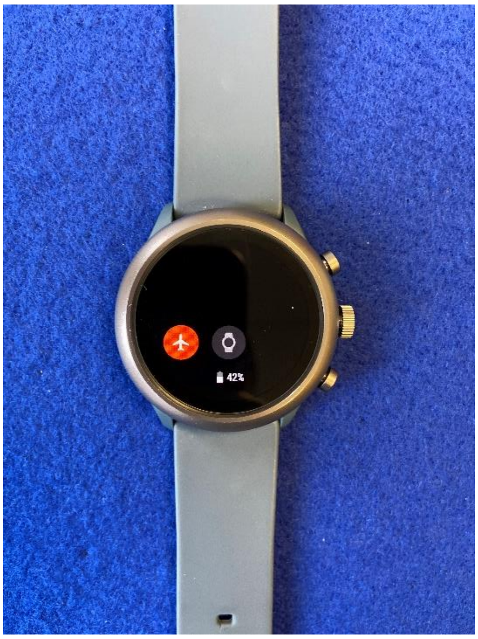

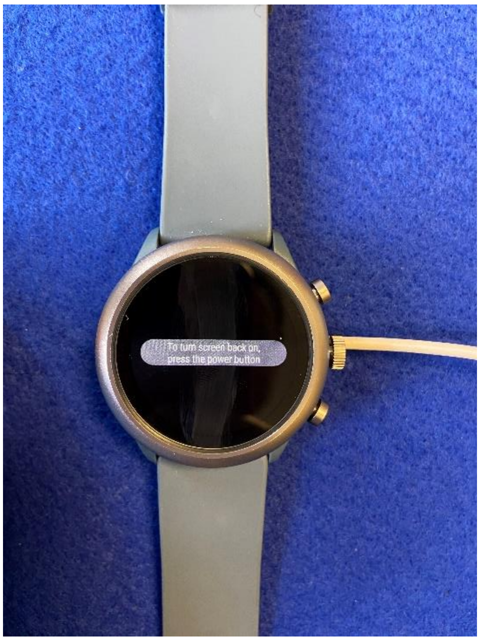

Figure 5. Aeroplane icon and sleep mode (accessed by swiping down from the Home screen)

3. The *Watch Your Steps* app

Overview

The study app is set up to prompt you to complete tasks at set times of the day and week. At these times, you will get a notification: the watch will buzz and give you instructions (Figure 6 – left image). You can also see any outstanding tasks and report any symptom at any time by opening the *Watch Your Steps* app using buttons A or B (Figure 6 – right image).

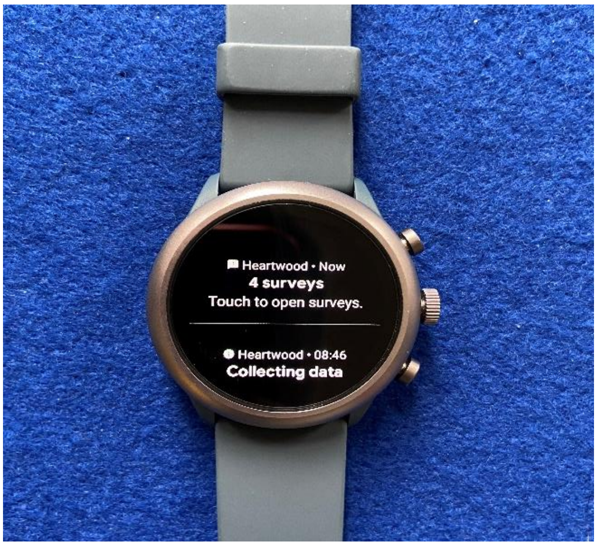

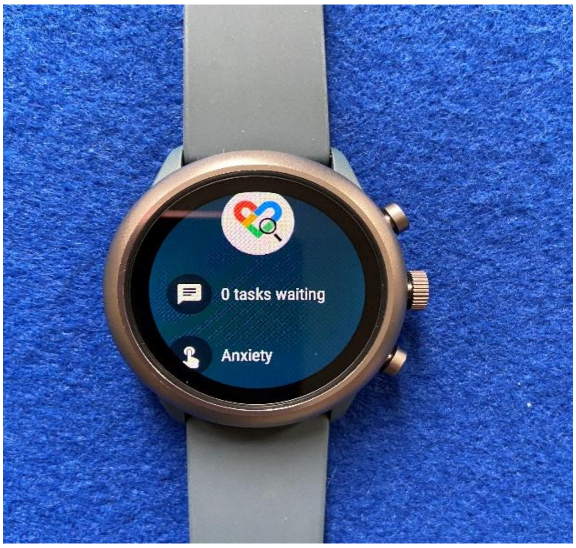

Figure 6. Notifications appear on the watch screen when surveys and tasks are due (left), or you can open the *Watch Your Steps* app to see a list of tasks (right)

Prompted tasks (Fig. 6 – left)

When tasks are scheduled to be completed, the watch will buzz and the screen will show you that a task is available to complete. Tasks include 'surveys' and 'active tasks', described in more detail in Sections 4 and 5 below.

- Different surveys and active tasks are requested at different times with varying frequency.
- The frequency of prompted tasks has been set by the research team.
- Don't worry if you miss surveys or want to add in another response. You are also able to answer any of the surveys or complete any of the active tasks whenever you wish via the app.

Accessing the tasks via the app (Fig. 6 – right)

To open the app, press button A or B from the Home screen. The app will show:
1. The number of outstanding tasks waiting to be completed
2. The full set of surveys and active tasks. You can scroll through these and answer any of them whenever you want

You may not have seen all of the questions as notifications. This is because some of the questions are specific to particular conditions. If you don't haven't stated that you have a stomach or bowel problem, for example, you won't normally be asked about bowel movements.

4. Completing tasks

There are two types of tasks the *Watch Your Steps* app will ask you to do. These are **surveys** and **active tasks**.

How to answer surveys

Surveys are questions that the watch will ask you about your symptoms or other information. There are two ways of responding to the survey: **an input wheel** or **discrete categories**. We have images below showing how to use both.

Surveys using the input wheel (Figure 7)

- Read the question, then scroll down to see the input wheel where you can record your response.
- Choose your answer by swiping the yellow handle around the face of the watch.
- The icon changes from a smile at the good end of the scale to a grimace at the end of the scale.
- When the yellow handle is at either end of the wheel, additional words describe the severity of that symptom.
- When you're done, the symptom level is shown as a figure above the wheel, press the 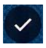 button in the middle of the screen to enter your response.

As an example, this is how to complete the 'Anxiety' survey:

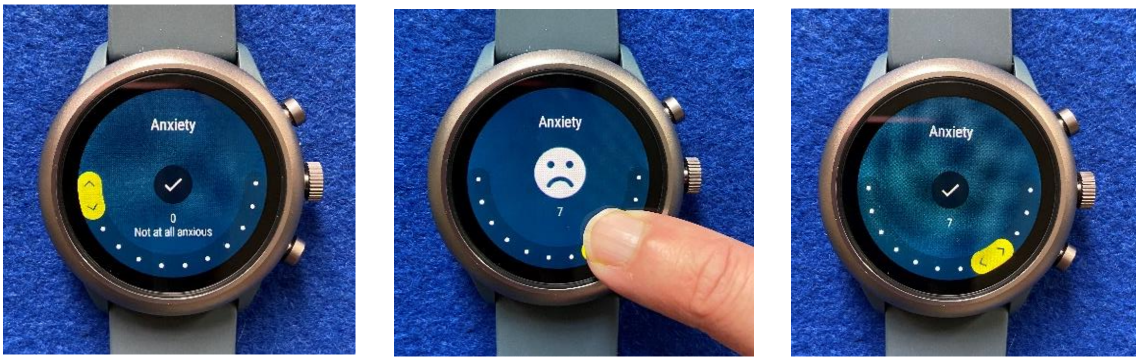

Figure 7. Entering responses using the input wheel

Discrete categories

- Read the question, then scroll down to see the possible categories of response
- Select the best response by tapping on the relevant circle
- Submit your answer by scrolling down to the bottom of the screen to press the button.

As an example, this is how to complete the ‘Appetite’ survey:

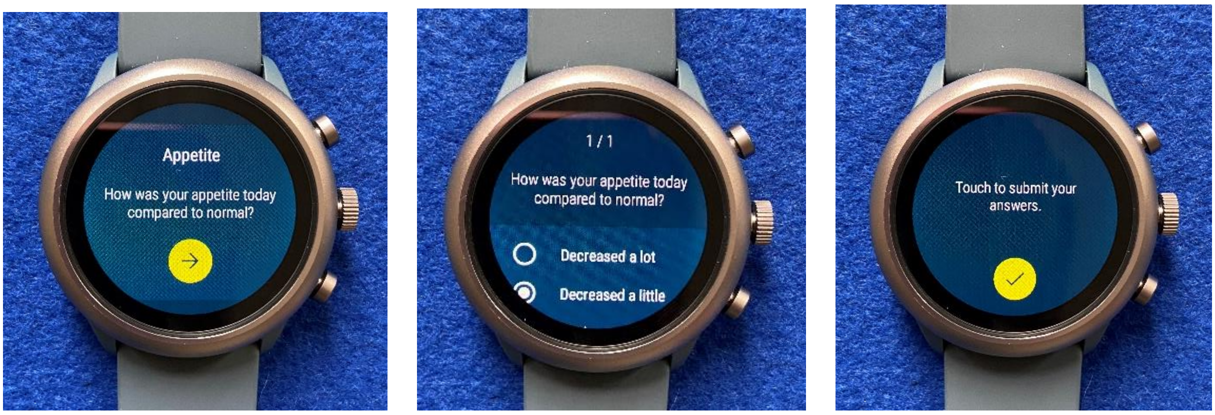

Figure 8. Entering responses using discrete categories

5. Understanding surveys and active tasks

How will I know when I need to complete surveys and tasks?

New alert notifications on the smartwatch
As described in Section 2, when new symptom surveys and active tasks are ready to complete, the watch will do two things:

(1) lightly buzz on your wrist
(2) the watch face will show you that a task is available by the white dot at the bottom of the screen

Outstanding tasks: when the app is open you will also see the number of outstanding tasks to complete (Figure 6, right), simply tap the survey alert to start it.

Missed alerts: if you miss an alert, you can always check the *Watch Your Steps* app by pressing Button A or B to see how many tasks there are waiting.

- If you don't answer surveys within a certain time frame, they will disappear.
- Don't worry, you'll receive new survey alerts the following day. And you can answer any question at any time via the app screen (Figure 6, right).

Which surveys will I have to complete?

These are chosen for you based on the answers that you give to two surveys the first time you use the *Watch Your Steps* app (when the watch and Mi-Fi is set up for you). These surveys will ask if you work and the type of long-term conditions that you manage: your responses will then inform the surveys that are scheduled on your app by us during the watch set-up.

There are also 'general' symptom surveys. These questions ask about 'general symptoms' that all people with different long-term conditions experience to different levels, such as: sleep quality, wellbeing, pain, mood, fatigue, stress and function.

There are also daily 'specific' long-term condition surveys. For example – those with joint problems will be given surveys on joint pain, those with gastrointestinal problems will be given surveys on bowel function, and so on.

Surveys are scheduled to be triggered in relation to the question asked. For instance, 'how would you rate your quality of sleep?' is triggered at 8am daily.

How do I complete active tasks?

Your watch will track your steps and body movement, rotation, direction, changes in altitude, and heart rate. This will happen throughout the day without you doing anything.

As well as this, there are active tasks that will appear on the watch to be completed, including a sit-stand test, walk test and tap test. Everyone taking part in the study will receive prompts to complete active tasks several times per week. Follow the instructions, and the watch sensors will record your activity. An example of the tap test is shown in Figure 9, and a full set of instructions for these three tests is shown in Table 1 below.

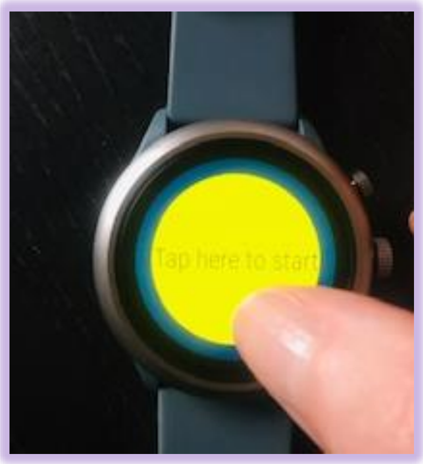

Figure 9. Start of the tap test active task

The active tasks have been selected to be simple and not stressful. Follow the instructions on the watch, and the watch sensors will record your activity. You can decline to do these, or abandon attempts at any time.

If you find the task physically challenging then please do not perform the active task.

What should I do if I cannot perform the active task?

If you prefer not to do the active task, you can tell us this on the watch by pressing ‘prefer not to do’ this takes you back to the app. You can also tell us if you struggled doing the active task a second time by pressing the relevant response: ‘I did the test’ (tick icon), ‘try again’ (try again icon) or ‘struggled, cancelled’ (X icon)

Table 2: Active tasks to do while wearing the watch following instructions on the app

Active task name	Instruction
Sit-Stand Test	'For this test you will be asked to sit down in a chair, fold your arms across your chest, then stand up and sit down twice'.<br>- Press 'Start' (press play icon) or 'Prefer not to do' (press X icon)<br>Start the task: 'Tap here and start standing up' (tap screen)<br>'Stand up and Sit down twice, and tap here' (tap screen)<br>- Press either: 'I did the test' (tick icon), 'try again' (try again icon) or 'struggled, cancelled' (X icon)
Tap test	'How many times you can tap the screen in 5 seconds?'<br>-Press 'Start' or 'Prefer not to do'.<br>-To the test: 'Press here to start'<br>-Numbers show up on screen per tap<br>-Final score shows up for you to tap to submit<br>-Press 'I did the test', 'try again' or 'struggled, cancelled'.
Walk Test	'For this test you will be asked to take 10 steps in a straight line, turn around and walk 10 steps back. You should walk however feels natural'.<br>- Press 'Start' or 'Prefer not to do' (takes you back to app)<br>Start the task: 'Tap here and start walking'<br>'Take 10 steps, turn, 10 steps back and tap here'<br>- Press 'I did the test', 'try again' or 'struggled, cancelled'.

6. Frequently Asked Questions

What is the battery life on the smartwatch?

The battery should give you a full day's worth of charge, although this does vary according to use. Please make sure you recharge the watch overnight using its own magnetic charger plugged in to the USB socket that the Mi-Fi device is plugged in (see charging section of this guide for detail).

How long does the battery take to charge?

It will take 60-90 minutes to fully charge the watch.

Should I continue to use the smartwatch if abroad, or on holiday?

If you are going beyond the UK for less than 5 days you should leave your MiFi device at home but take the watch charger and the watch with you. The watch can store up to five days of data and will send this when you get home and connect with the Mi-Fi. Please ensure you still take the charger and smartwatch with you in order to continue to take part in the study. You may also need to use your own international power adapter if going abroad in order to charge your watch.

If you are going away for longer than 5 days, please just leave your watch at home.

What happens if the smartwatch app appears stuck, how do I submit my smartwatch data then?

First, try rebooting the app by pressing button C for 20 seconds. If this doesn't work, take a photo of the watch screen and email this to us at watchyoursteps@manchester.ac.uk

When should I put the smartwatch on?

Put the watch on as soon as you get up in the morning (fully charged).

When should I take the smartwatch off?

Remove the watch at the end of the day just before you go to bed, or when the battery runs out. If your battery runs out before the end of the day, you should recharge then put the watch back on until the end of the day.

Can I wear the watch in water, or while playing sports?

It is safe to keep the watch on your wrist when you are swimming, running, jogging and taking a shower. The watch is water resistant only and is safe to be worn when swimming and doing other sports.

How can I avoid damage to the watch?

Do not expose watch to extremely high or low temperature, direct sunlight for extended period and high water pressure.

How do I send data from the watch to the study team?

Data will be sent automatically when connected to the charger by linking wirelessly to the Mi-Fi device. The MiFi device and the watch charger should both be plugged into the twin USB charger provided. Data transfer may take some time if data is large. This process of data handling, security, privacy and transfer are explained fully in the Participant Information Sheet.

I want to withdraw from the study, what should I do?

You can withdraw from the study at any time and you don't have to give a reason. Please email watchyoursteps@manchester.ac.uk and we will arrange for the return of the smartwatch. If you *are* happy to, please do give us feedback as to why you wanted to withdraw. So we can learn how to improve smartwatch studies like this.

When will I have to answer the various questions?

You will get prompts to answer surveys multiple times per day. Not all questions will come at the same frequency as others. You will get several active tasks to complete each week.

What happens if I don't answer a question?

Notifications will remain on the watch for a set period of time. If you haven't managed to answer the questions in time, the notification will disappear and you will be able to answer the next questions you receive. You can always answer any question at any time via the app.

While we appreciate that you may not be able to answer every question, just do your best to answer as many as you can.

Contact details

If you have any queries about this user guide or a concern with your smartwatch then please email the University project team: watchyoursteps@manchester.ac.uk

Or call the researcher Nicola Small direct on: **0161 275 1671**

Appendix: Full list of surveys, accessible at any time via the app screen

**Number of surveys, name of survey**	**Question** (within each survey)	**Answers**
Anxiety	How anxious were you today?	0 = Not at all anxious<br>10 = Extremely anxious
Appetite	How was your appetite today compared to normal?	- Decreased a lot<br>- Decreased a little<br>- Unchanged<br>- Increased a little<br>- Increased a lot
Average breathlessness	How was your breathlessness on average today?	0 = No breathlessness<br>10 = Extreme
Average itch severity	How was your itch, on average, in the past 24 hours?	0 = No itch<br>10 = Worst imaginable
Bowel movements	How were your bowel movements today?	- No bowel movement<br>- Hard lumps, hard to pass/ Severe constipation;<br>- Sausage shaped but lumpy/ Mild constipation;<br>- Sausage shaped, surface cracks/ Normal<br>- Like a sausage, smooth and soft/ Normal<br>- Soft blobs, clear cut edges/ Lacking fibre<br>- Fluffy pieces with ragged edges/ Mild diarrhoea<br>- Watery, no solid pieces/ Severe diarrhoea
Drowsiness	Please select the number that best describes your drowsiness NOW (feeling sleepy)	0 = no drowsiness<br>10 = worst possible
Fatigue	How much fatigue do you feel?	0 = No fatigue<br>10 = Extreme fatigue
Function	How much difficulty have you had with daily tasks today?	0 = No difficulty<br>10 = Extreme difficulty

Hours missed health	How many hours did you miss from work because of health this week?	- None<br>- Less than 1 hour<br>- 1-2 hours<br>- 2-5 hours<br>- 5-10 hours<br>- 10-15 hours<br>- 15-20 hours<br>- 20-25 hours<br>- 25-30 hours<br>- 30-40 hours<br>- 40+ hours
Hours missed other	How many hours did you miss from work because of any other reason, such as holidays, this week?	- None<br>- Less than 1 hour<br>- 1-2 hours<br>- 2-5 hours<br>- 5-10 hours<br>- 10-15 hours<br>- 15-20 hours<br>- 20-25 hours<br>- 25-30 hours<br>- 30-40 hours<br>- 40+ hours
Hours worked	How many hours did you work this week?	- None<br>- Less than 1 hour<br>- 1-2 hours<br>- 2-5 hours<br>- 5-10 hours<br>- 10-15 hours<br>- 15-20 hours<br>- 20-25 hours<br>- 25-30 hours<br>- 30-40 hours<br>- 40+ hours
Mood	How is your mood?	0 = Very low<br>10 = Very happy
Morning stiffness	How long were you stiff for this morning?	- Not at all<br>- 0-29 minutes<br>- 30-59 minutes<br>- 1-2 hours<br>- 2-4 hours

		- Over 4 hours
Pain level	How was your overall level of pain today?	0 = No pain<br>10 = Worst possible pain
	What is your pain level now?	0 = No pain<br>10 = Worst possible pain
Productivity	How much did your health affect your productivity at work this week?	0 = No effect on work<br>10 = Unable to work
Sleep Quality	How would you rate your sleep quality?	0 = Terrible<br>10 = Excellent
Stress	How stressed are you?	0 = Not at all<br>10 = Extremely
Wellbeing	How well did you feel today?	0 = Very well<br>10 = Very unwell
Worst breathlessness	How was your worst breathlessness in the past 24 hours?	0 = None<br>10 = Extreme breathlessness
Worst itch severity	How was your worst itch in the past 24 hours?	0 = no itch<br>10 = worst imaginable
	How was your itch, on average in the past 24 hours?	0 = no itch<br>10 = worst imaginable itch
Health Survey	Mobility	- I have no problems in walking about<br>- I have slight problems in walking about<br>- I have moderate problems in walking about<br>- I have severe problems in walking about<br>- I am unable to walk about
	Self-care	- I have no problems washing or dressing myself<br>- I have slight problems washing or dressing myself<br>- I have moderate problems washing or dressing myself<br>- I have severe problems washing or dressing myself<br>- I am unable to wash or dress myself

	Usual activities	- I have no problems doing my usual activities<br>- I have slight problems doing my usual activities<br>- I have moderate problems doing my usual activities<br>- I have severe problems doing my usual activities<br>- I am unable to do my usual activities
	Pain/ discomfort	- I have no pain or discomfort<br>- I have slight pain or discomfort<br>- I have moderate pain or discomfort<br>- I have severe pain or discomfort<br>- I have extreme pain or discomfort
	Anxiety/ depression	- I am not anxious or depressed<br>- I am slightly anxious or depressed<br>- I am moderately anxious or depressed<br>- I am severely anxious or depressed<br>- I am extremely anxious or depressed
	How good or bad is your health today?	0 = Worst possible health<br>100 = Best possible health
