# Supplementary material for: Engagement with consumer smartwatches for tracking symptoms of individuals living with multiple long-term conditions (multimorbidity): A longitudinal observational study
Source: J Multimorb Comorb. 2021 Nov 30;11:26335565211062791. doi: 10.1177/26335565211062791 (PMC8637784; doi:10.1177/26335565211062791)
Supplement: sj-pdf-2-cob-10.1177_26335565211062791 – Supplemental Material for Engagement with consumer smartwatches for tracking symptoms of individuals living with multiple long-term conditions (multimorbidity): A longitudinal observational study [file sj-pdf-2-cob-10.1177_26335565211062791.pdf]

Baseline questionnaire

Record ID

____

Study ID

____
(To be provided by the study team)

What is your current age?

- ◯ 18-29
- ◯ 30-39
- ◯ 40-49
- ◯ 50-59
- ◯ 60-69
- ◯ 70-79
- ◯ 80-89
- ◯ 90 or older

What is your gender?

- ◯ Male
- ◯ Female
- ◯ Non-binary/ third gender
- ◯ Prefer not to say
- ◯ Prefer to self-describe

Please enter the first half of your postcode

____
(This information will not be used beyond research team or research purpose)

What is your ethnicity?

- ◯ White
- ◯ Mixed ethnic group
- ◯ Asian or Asian British
- ◯ Black or Black British
- ◯ Other
- ◯ Prefer not to answer

(This information will not be used research team or research purpose)

Do you have a long-term health condition in any of the following areas?

- ☐ Bone, joint and muscle (e.g., arthritis, neck/back pain, chronic pain)
- ☐ Skin (e.g., psoriasis, eczema)
- ☐ Heart and lung (e.g. angina, heart failure, COPD/ asthma)
- ☐ Stomach and bowel (e.g., persistent nausea and vomiting, inflammatory bowel disease)
- ☐ Kidney (e.g., chronic kidney disease)
- ☐ Endocrine (e.g., diabetes, thyroid disorders)
- ☐ Mental health (e.g., anxiety, depression, schizophrenia)
- ☐ Neurological (e.g., epilepsy, MS, Parkinsons)
- ☐ Other

(Tick all that apply)

Do you use a walking aid?

- ☐ Stick
- ☐ Crutch
- ☐ Walking frame
- ☐ Mobility scooter
- ☐ Wheelchair
- ☐ No walking aid

Do you take part in any regular low or moderate intensity exercise (e.g., walking)?	◯ Yes<br>◯ No
Do you do any vigorous-intensity sports, fitness or recreational (leisure) activities that cause large increases in breathing or heart rate (e.g., running or football)?	◯ Yes<br>◯ No
Do you own any activity monitoring devices?	☐ Wearable technology (e.g., fitbit)<br>☐ Pedometer<br>☐ Smartphone activity monitoring app<br>☐ Smartwatch activity monitoring app<br>☐ I do not use an activity monitoring device<br>(Tick all that apply)
Do you use any smartphone health/well-being apps?	◯ Yes<br>◯ No
How frequency do you use any smartphone, smartwatch health/wellbeing apps?	◯ Always<br>◯ Often<br>◯ Sometimes<br>◯ Rarely<br>◯ Never
Please indicate how strongly you agree with the following statement:<br>The use of smartphone and smartwatch health/wellbeing apps affect how you manage your condition/health?	◯ Strongly agree<br>◯ Agree<br>◯ Neither agree nor disagree<br>◯ Disagree<br>◯ Strongly disagree
Would you be willing to be interviewed by a member of the research team about your experiences with long term conditions and using smartwatch?<br><br>Note: Interviews will take place at the beginning and end of the study and will take around an hour each. You may be required to make additional visits to the University for these. Travel expenses will be paid, along with a High Street shopping voucher to compensate for your time (at a rate of £10 per interview)	◯ Yes<br>◯ No
