# Supplementary material for: Engagement with consumer smartwatches for tracking symptoms of individuals living with multiple long-term conditions (multimorbidity): A longitudinal observational study
Source: J Multimorb Comorb. 2021 Nov 30;11:26335565211062791. doi: 10.1177/26335565211062791 (PMC8637784; doi:10.1177/26335565211062791)
Supplement: sj-pdf-3-cob-10.1177_26335565211062791 – Supplemental Material for Engagement with consumer smartwatches for tracking symptoms of individuals living with multiple long-term conditions (multimorbidity): A longitudinal observational study [file sj-pdf-3-cob-10.1177_26335565211062791.pdf]

Watch Your Steps - Self-administered End-of-Study Questionnaire

The following questionnaire will ask about your experience of participating in Watch Your Steps. You will be asked X questions, grouped into questions about wearing the watch, how easy it was to answer the questions on the watch, and finally a few more general questions. It should take about 5 minutes to complete [check]

Thank you for taking part!

Watch/Hardware

		Always	Often	Sometimes	Rarely	Never
1)	How frequently did you wear the watch during the study?	○	○	○	○	○

2) If you did take the watch off, when was this? Please select all that apply

- ☐ Charging
- ☐ Showering
- ☐ Bathing
- ☐ Swimming
- ☐ At the gym
- ☐ Cooking
- ☐ At work
- ☐ In bed
- ☐ Other, please specify

		Strongly agree	Agree	Neither agree nor disagree	Disagree	Strongly disagree
3)	I often forgot to wear the watch after charging	○	○	○	○	○
4)	The watch stopped me from going about my normal activities during the day	○	○	○	○	○
5)	I found the watch to be comfortable to wear	○	○	○	○	○
6)	I often forgot to charge the watch	○	○	○	○	○
7)	Charging the battery every day was easy to manage	○	○	○	○	○

		Strongly agree	Agree	Neither agree nor disagree	Disagree	Strongly disagree
8)	The battery lasted the full day	○	○	○	○	○

9) Any other comment about wearing the watch? ______________________________

Watch/Software

		Strongly agree	Agree	Neither agree nor disagree	Disagree	Strongly disagree
10)	It was easy to navigate through the smartwatch app	○	○	○	○	○

		Strongly agree	Agree	Neither agree nor disagree	Disagree	Strongly disagree
11)	I found the wording of the symptom questions easy to understand	○	○	○	○	○
12)	I found it easy to understand the scale and how to report the severity of my symptoms	○	○	○	○	○

13) Do you have any comment about wording of symptom questions and ease in reporting symptoms? ________________________________

Data Entry

		Strongly agree	Agree	Neither agree nor disagree	Disagree	Strongly disagree
14)	I found it easy to enter my manual symptoms data into the watch app	○	○	○	○	○

15) Any comments regarding ease of entering manual symptoms data? ________________________________

		Much too high	A bit too high	About right	A bit too low	Much too low
16)	Overall, I found the total number of questions per day________	○	○	○	○	○
17)	Was the frequency of the questions (how many times per day the questions were asked) ________?	○	○	○	○	○

18) Which of the following symptoms were useful to track?

- ☐ Pain
- ☐ Mood
- ☐ Well-being
- ☐ Fatigue
- ☐ Sleep
- ☐ Stress
- ☐ Function

(Tick all that apply)

19) Which of the following symptoms would you prefer not to have had to respond to?

- ☐ Pain
- ☐ Mood
- ☐ Well-being
- ☐ Fatigue
- ☐ Sleep
- ☐ Stress
- ☐ Function

(Tick all that apply)

20) Any additional comment about the symptoms?

____

	Strongly agree	Agree	Neither agree nor disagree	Disagree	Strongly disagree
21) I found the timing of the 8 am questions convenient for me	○	○	○	○	○
22) I found the timing of the 12 pm questions convenient for me	○	○	○	○	○
23) I found the timing of the 4 pm questions convenient for me	○	○	○	○	○
24) I found the timing of the 6 pm questions convenient for me	○	○	○	○	○
25) I found the timing of the 8 pm questions convenient for me	○	○	○	○	○

26) I would have liked to report symptoms after 8 pm

- ○ Yes
- ○ No

27) If yes, then which symptom(s)?

- ☐ Pain
- ☐ Mood
- ☐ Well-being
- ☐ Fatigue
- ☐ Sleep
- ☐ Stress
- ☐ Function

(Skip question if you would not have liked to report symptoms after 8 pm)

	Strongly agree	Agree	Neither agree nor disagree	Disagree	Strongly disagree
28) I reported symptoms at other times when not prompted	○	○	○	○	○
29) I found the ability to report symptoms at other times useful	○	○	○	○	○
30) I found the prompts to complete the questions disruptive	○	○	○	○	○

Other

	Strongly agree	Agree	Neither agree nor disagree	Disagree	Strongly disagree
31) Using the MiFi device was easy	◯	◯	◯	◯	◯
32) I would participate in a similar study again, if I had the opportunity	◯	◯	◯	◯	◯

33) Is there anything that could have made your participation in this study more useful or easy for you?

____
(Please help us to make user experience better in future studies)

34) Are there any changes you would suggest making to the watch?

____

35) Do you think the use of smartphone, smartwatch health/wellbeing apps affect how you manage your condition/health?

- ◯ Strongly agree
- ◯ Agree
- ◯ Neither agree nor disagree
- ◯ Disagree
- ◯ Strongly disagree

36) Is there a reason as to why you preferred not to do an active task on the smartwatch?

- ◯ Feeling unwell
- ◯ On holiday (e.g. not doing daily routine)
- ◯ Too busy
- ◯ No reason
- ◯ Other

37) Is there anything that limited your ability to record daily symptoms?

- ◯ Feeling unwell
- ◯ On holiday (e.g. not doing usual routine)
- ◯ Too busy
- ◯ Nothing
- ◯ Other

38) Were there any long periods of time where you were unable to enter your response to either an active task or symptom data on the smartwatch? (E.g. a couple of days, a week, month).

- ◯ Yes
- ◯ No

39) Can you tell us why?

____
